# DENND5B Gene Expression as a Trigger for the Development of Diabetes Mellitus–Peripheral Artery Disease: Insights from a Univariate and Multivariate Mendelian Randomization Study

**DOI:** 10.5334/gh.1373

**Published:** 2024-12-05

**Authors:** Qiaoqiao Li, Fuli Cao, Xueping Gao, Yuan Xu, Bo Li, Tianyang Hu

**Affiliations:** 1 Department of Cardiology, The Second Affiliated Hospital of Chongqing Medical University, No. 76 Linjiang Road, Chongqing, 400010, China; 2 Department of Geriatrics and Special Service Medicine, First Affiliated Hospital of Army Medical University (Southwest Hospital), Chongqing, 400038, China; 3 Department of Laboratory Medicine, The First Affiliated Hospital of Chongqing Medical University, Chongqing, 400042, China; 4 Department of Pathology, Zhongshan Hospital Fudan University, Shanghai, 200000, China; 5 Chongqing University Jiangjin Hospital, Chongqing University, Chongqing, 402260, China; 6 Precision Medicine Center, The Second Affiliated Hospital of Chongqing Medical University, Chongqing, 400010, China

**Keywords:** Diabetes combined peripheral artery disease, Summary-data-based Mendelian randomization, Multivariable mendelian randomization

## Abstract

**Background:**

Peripheral artery disease (PAD) is a manifestation of systemic atherosclerosis that can result in limb pain, disability, or mortality. Notably, diabetes mellitus (DM) stands out as one of the most significant risk factors for the development of PAD. Compared to individuals with PAD but no DM, those with concurrent DM and PAD (DM-PAD, diabetes mellitus–peripheral artery disease) face a seven-fold higher risk of critical limb ischemia and a five-fold higher risk of amputation. However, the pathogenic factors and effective therapeutic targets for DM-PAD still remain elusive.

**Method:**

To identify candidate hub genes and develop insights into the pathogenesis of DM-PAD, we employed a comprehensive approach encompassing two-sample Mendelian Randomization (two-sample MR), summary data-based Mendelian randomization (SMR), and Bayesian colocalization (COLOC) methods. These methodologies facilitated the integration of summary-level data derived from genome-wide association studies of DM-PAD with expression quantitative trait locus (eQTLs) studies conducted on blood samples.

**Result:**

*DENND5B, C4A*, and *CYP21A2* were found to have passed two-sample MR and SMR analyses, indicating their status as hub genes associated with DM-PAD through mechanisms involving not linkage but rather causality. The COLOC analysis provided strong evidence suggesting that *DENND5B* and the DM-PAD trait were influenced by the common causal variant rs1150948.

**Conclusion:**

Our study has pinpointed several crucial genes (*DENND5B, C4A*, and *CYP21A2*), notably the *DENND5B* gene, as potential regulators in the pathogenesis of DM-PAD. These discoveries hold promises for shedding light on the underlying mechanisms and novel targets of the disease in future research.

## Introduction

Peripheral arterial disease (PAD) is a clinical manifestation of atherosclerosis that can result in the obstruction of blood flow. This obstruction can occur due to various factors such as embolism, thrombosis, or the narrowing of peripheral arteries (1). A recent comprehensive review estimated that an astonishing number exceeding 200 million individuals worldwide are afflicted by PAD, which represents a substantial increase over the previous decade (2). Moreover, those with symptomatic PAD face an elevated risk of cardiovascular morbidity and mortality, which, in turn, is accompanied by a detrimental impact on their overall quality of life (3). A series of risk factors, including smoking, obesity, hypertension, dyslipidemia, diabetes mellitus (DM), aging, a family history of peripheral artery disease, as well as heart disease or other cardiovascular ailments, significantly contribute to the development of PAD (4). Notably, DM constitutes a prominent and formidable risk factor for the development of PAD (4,5). In diabetic patients, it is remarkable that PAD progresses rapidly, manifests a more diffuse pattern, and predominantly affects the distal limb arteries, encompassing the tibial and peroneal arteries, in stark contrast to non-diabetic PAD (6,7). Furthermore, estimations reveal that individuals with PAD concurrent with DM (DM-PAD) exhibit a seven-fold higher risk of critical limb ischemia and a five-fold higher risk of amputation, when compared to patients with PAD but no DM (8). However, the pathophysiological mechanisms underlying DM-PAD are intricate and multifaceted. Consequently, the identification of additional pathogenic genes has emerged as a pivotal aspect in the prevention and treatment of DM-PAD.

Mendelian randomization (MR) is a method that can infer causal relationships between two heritable complex traits from observational studies (9,10). Previous Mendelian randomization studies have reported the detrimental effects of smoking and type 2 diabetes (T2D) on PAD, while the effects of low-density lipoprotein cholesterol and triglycerides were not confirmed. Additionally, lower education levels and insomnia have been identified as novel risk factors for PAD (11). In addition, Xiu et al. (12) have utilized the enrichment of complex trait loci for the expression quantitative trait loci (eQTLs) to demonstrate the causal link between specific molecules and PAD. Their discoveries underscore the significance of vigilantly monitoring the PAD status in individuals with T2D and propose novel genetic biomarkers for evaluating the risk of PAD among T2D patients. However, previous studies in this field have still deficiently addressed the presence of DM-PAD. Further exploration is needed to identify new genetic targets associated with DM-PAD.

In our study, we utilized the SMR method (13) and Bayesian colocalization (COLOC) analysis (14) to integrate summary-level data from the genome-wide association studies (GWAS) on DM-PAD with expression quantitative trait locus (eQTLs) data obtained from blood samples. Our goal is to identify the trigger genes associated with DM-PAD, facilitating future investigations into the disease’s underlying mechanisms.

## Methods

### Data sources for the exposure and outcome in the Mendelian randomization studies

We identified eQTLs that were statistically significant, with a *p*-value of <5 × 10^–8^, from the eQTLGen Consortium, a meta-analysis of eQTLs in peripheral blood samples collected from 31,684 individuals (15). Data on body mass index (BMI; GWAS ID: ieu-b-40), low-density lipoprotein cholesterol (LDL-C; GWAS ID: ieu-b-110), high-density lipoprotein cholesterol (HDL-C; GWAS ID: ieu-b-109), and triglyceride (TG; GWAS ID: ieu-b-111) were obtained from the IEU open GWAS database (https://gwas.mrcieu.ac.uk/datasets/). Furthermore, we obtained the genetic associations of instrumental variables with DM-PAD from European cohorts, specifically the FinnGen study (Release 7). The dataset for the DM-PAD outcome comprised 11,197 cases of individuals affected by DM-PAD and 225,597 controls. All the related GWAS summary data and materials have been made publicly available and comprehensive information regarding data sources is presented in Table 1. The schematic illustration was drawn using Figdraw tools (www.figdraw.com).

**Table 1:** Characteristics of selected genome-wide association studies.


OUTCOMES	CONSORTIUM	SAMPLE SIZE (CASES/CONTROLS)	POPULATION	UNIT

DM-PAD	NA	11,197/225,597	European	Event

eQTLs	eQTLGen	31,684	European	NA

BMI	GIANT	681,275	European	NA

Triglycerides	UK Biobank	441,016	European	NA

HDL cholesterol	UK Biobank	403,943	European	NA

LDL cholesterol	UK Biobank	173,082	European	NA


DM-PAD, diabetes mellitus-peripheral artery disease; eQTLs, expression quantitative trait locus; BMI, body mass index; HDL, high-density lipoprotein cholesterol; LDL, low-density lipoprotein cholesterol.

### SMR analysis

The SMR method, a summary-data-based MR method, investigates the association between the expression level of a gene and the outcome of interest using summary-level data from genome-wide association studies (GWAS) and eQTL studies (13). This method can generate effect estimates in a Mendelian randomization (MR) study by employing expression quantitative trait loci (eQTLs) within a 2000 kb window as instrumental variables (cis-eQTL) (13). Allele harmonization and analysis were conducted using the SMR software version 1.03 (https://cnsgenomics.com/software/smr/#Overview). For the SMR tests, false discovery rate (FDR) <0.05 was considered statistically significant. For the HEIDI test, a *p*-value >0.01 was considered significant, indicating that the observed association was not due to linkage (16,17).

### Two-sample Mendelian randomization (two-sample MR) analysis

Quality control measures were taken in a two-sample Mendelian randomization study examining the association between genes and the risk of DM-PAD to ensure accurate and precise causal inference (18). Firstly, instrumental variables (eQTLs) were selected based on a *p*-value below the genome-wide significance threshold (5 × 10^–8^). Secondly, an aggregation process was conducted to assess linkage disequilibrium (LD) between the instrumental variables, using a clumping distance of 10,000 kb and *r*^2^ < 0.001 as the criteria. Thirdly, palindromic SNPs were excluded as instrumental variables. Fourthly, instrumental variables with *F*-statistic <10 were considered weak and excluded. The *F*-statistic, calculated using the formula [(*n* – *k* – 1)/*k*] × [*R*2/(1 – *R*2)], measures the strength of the relationship between the instrumental variables and the exposure (19). Here, *R2* represents the proportion of variation explained by the SNPs in the exposure. and R2 was calculated using the formula *R2* = (2 × EAF × (1 – EAF) × Beta2)/[(2 × EAF × (1 – EAF) × Beta2) + (2 × EAF × (1 – EAF) × n × SE2)], where EAF stands for the effect allele frequency, Beta indicates the estimated genetic effect of SNP on exposure, EAF is effect allele frequency, SE is standard error of the estimated effect, and n denotes the sample size, and *k* indicates the number of instrumental variables.

For this Mendelian randomization study, the ‘TwoSampleMR’ package in R software version 4.1.0 was utilized to perform allele harmonization and analysis (20). Initially, the IVW-MR and Wald ratio methods were implemented to combine effect estimates of genetic variants associated with gene levels, by treating these genetic variants as instruments. The primary objective was to test and validate the genes that have been recognized as significant contributors to the progression of DM-PAD in the SMR analysis.

### Sensitivity analysis in two-sample MR analysis

To evaluate the effects of horizontal pleiotropy, the MR-Egger Intercept Test was employed. Additionally, Cochran’s Q statistic was utilized to measure the heterogeneity among the chosen SNPs (21).

### COLOC analysis

We used the ‘coloc’ package in the R software (version 4.2.1) to conduct COLOC analysis. This analysis enabled us to explore whether the single nucleotide polymorphism (SNP) associated with both gene expression and phenotype at the same genomic location were indeed shared causal variants. As a result, we could determine whether gene expression and phenotype were colocalized (14). To assess the hypotheses, COLOC analysis computes posterior probabilities (PPs) for five scenarios: (1) H0, no association with gene expression or phenotype; (2) H1, association with gene expression but not with phenotype; (3) H2, association with phenotype but not with gene expression; (4) H3, independent SNVs associated with both gene expression and phenotype; and (5) H4, shared causal SNVs associated with both gene expression and phenotype. A high PP for H4 (PP.H4 > 0.80) strongly indicates the presence of shared causal variants influencing both gene expression and phenotype (22). In our analysis, we utilized the coloc.abf function with default settings, assigning prior probabilities of 1 × 10^–4^ for H1 and H2, and 1 × 10^–5^ for H4. The analysis was conducted within a 1000 kb region on both sides of the lead variant with the smallest *p*-value in the GWAS data.

### Multivariable Mendelian randomization (MVMR) analysis

In order to examine whether genes served as an independent risk factor for DM-PAD, we conducted an MVMR analysis (23), which was adjusted for multiple confounding variables including BMI, LDL-C, HDL-C, and TG in our analysis.

## Results

### Performing SMR analyses to identify pathogenic genes for DM-PAD

**Figure 1:**
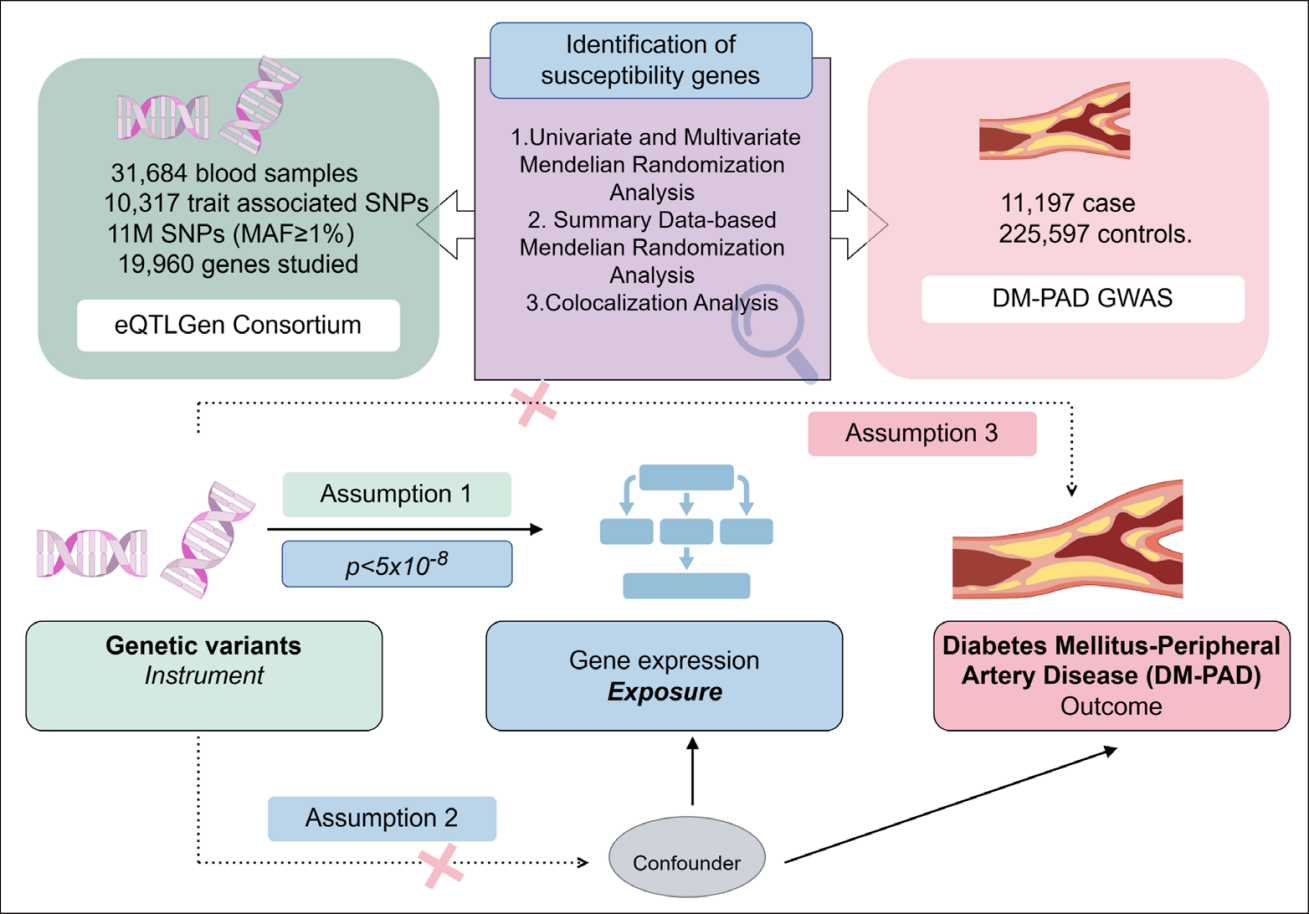
**Schematic diagram of MR analysis.** Assumption 1: Indicated by the solid line, the instrumental variants directly influence the expression of genes. Assumption 2: Represented by dashed lines, the instrumental variables are not associated with any potential confounders. Assumption 3: The instrumental variables affect the outcome solely through the exposure without any involvement in other causal pathways.

**Figure 2:**
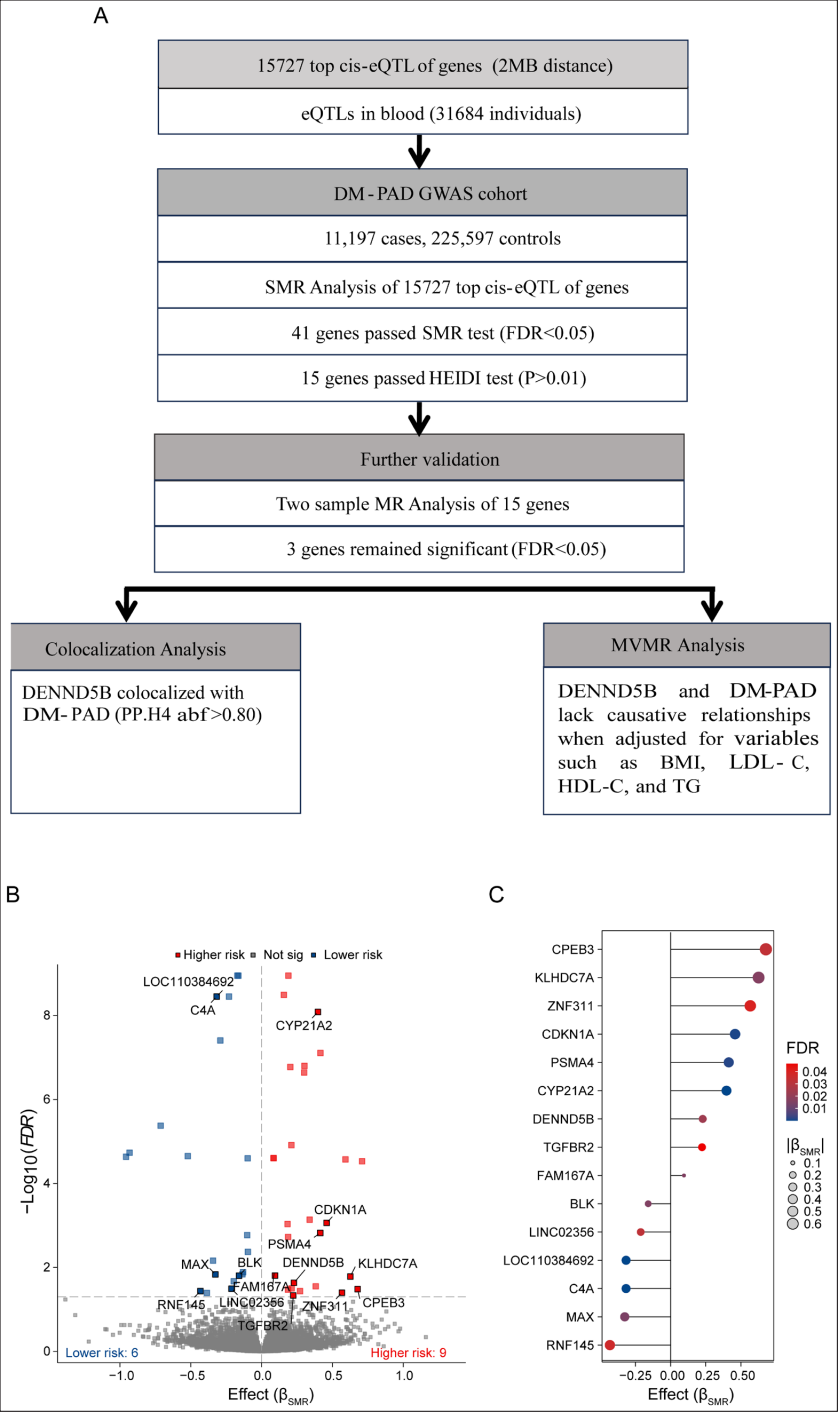
**Identification of candidate risk genes for DM-PAD by using SMR analysis. A.** Flowchart of the study. DM-PAD, diabetes mellitus–peripheral artery disease (SMR, summary-data-based Mendelian randomization; HEIDI, heterogeneity in dependent instruments; FDR, false discovery rates; MVMR, multivariable mendelian randomization). **B.** Volcano plot of causal effect between genes and DM-PAD: The abscissa represents b_SMR_, while the ordinate represents –log_10_(FDR). Red squares indicate high-risk candidate genes for DM-PAD, while blue squares represent low-risk candidate genes. Genes with black text labels represent those that passed the HEIDI test with a *p*-value >0.01. C. The dot-plot graph illustrates the impact of 15 risk candidate genes on DM-PAD, with |b_SMR_| values influencing the size of the dots and FDR determining their color (FDR, false discovery rate; DM-PAD, diabetes mellitus–peripheral artery disease).

Schematic representation of an MR analysis was illustrated (Figure 1) The detailed analysis process is shown in the flow chart (Figure 2A). We conducted an SMR analysis to integrate GWAS and blood cis-eQTL data, with the aim of identifying the genes whose expression in blood was significantly associated with the trait of DM-PAD. The resulting volcano map showed a total of 41 genes (represented by red and blue small squares) that passed the SMR test with a *p*-adjust (FDR) value below 0.05. However, only 15 of these genes (represented by small red and blue squares with genetic tags), including *PSMA4, CPEB3, CDKN1 A, MAX, BLK, RNF145, FAM167 A, TGFBR2, DENND5B, KLHDC7 A, ZNF311, CYP21A2, C4A, LOC110384692*, and *LINC02356*, passed the HEIDI test with a *p*-value >0.01 (Figure 2B; Supplementary Table 1). To provide more details, we created a lollipop chart that displayed the effect size (β) and FDR value of the 15 identified genes associated with DM-PAD. Out of these genes, six genes (*BLK, LINC02356, LOC110384692, C4A, MAX*, and *RNF1*) were found to have a protective effect for DM-PAD, as their β values were <0, while nine genes (*CPEB3, KLHDC7 A, ZNF311, CDKN1 A, PSMA4, CYP21A2, DENND5B, TGFBR2*, and *FAM167 A*) were considered to be dangerous, as their β values were >0 (Figure 2C).

### Two-sample MR analysis

**Figure 3:**
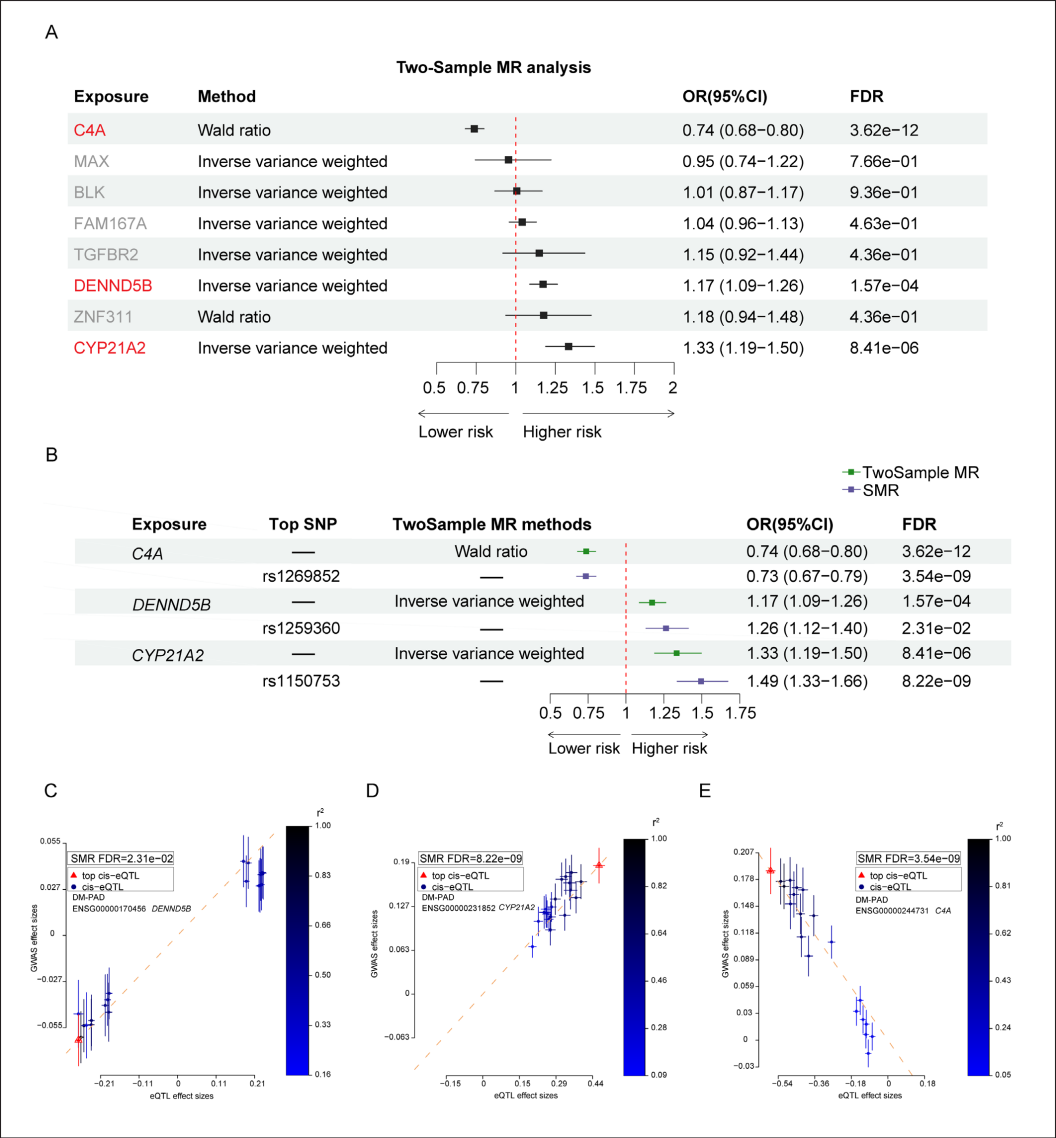
**Further exploration of risk genes for DM-PAD through the integration of SMR and two-sample MR analyses. A.** Forest plot showing the odds ratios (OR) for associations between candidate genes and DM-PAD risk in two-sample MR analysis. **B.** Forest plot illustrating the OR for the associations between *C4A, DENND5B*, and *CYP21A2* and the risk of DM-PAD in two-sample MR and SMR analysis. The FDR was calculated using the Benjamini–Hochberg method. **C–E.** Plotting effect sizes from DM-PAD GWAS against those from eQTL in *DENND5B* (C), *CYP21A2* (D), and *C4A* (E). The *r*^2^ value represents the linkage disequilibrium between the top cis-eQTL and other cis-eQTL. Red triangles represent the top cis-eQTL, while blue circles represent other cis-eQTL (OR, odds ratios; DM-PAD, diabetes mellitus–peripheral artery disease; SMR, summary data-based Mendelian randomization; FDR, false discovery rate; eQTL, expression quantitative trait locus).

Two-sample MR analysis was conducted to further investigate the association between 15 candidate genes and DM-PAD. The results showed that under the condition of quality control, only three genes (*C4A, CYP21A2*, and *DENND5B*) have passed the MR test with a *p*-adjust (FDR) value <0.05 (Figure 3A; Supplementary Table 2). Specifically, inverse-variance weighted (IVW) and Wald ratio analysis results revealed that a causal association between genetically predicted *CYP21A2*, and *DENND5B* and higher risk of DM-PAD were observed: *CYP21A2* (OR: 1.33; 95%CI: 1.19–1.50; IVW_FDR_ = 8.41 × 10^–6^), *DENND5B* (OR: 1.17; 95%CI: 1.09–1.26; IVW_FDR_ = 1.57 × 10^–4^), *C4A* and lower risk of DM-PAD were detected: *C4A* (OR: 0.74; 95%CI: 0.68–0.80; Wald ratio _FDR_ = 3.62 × 10^–12^; Figure 3B). Moreover, the *F* values of each SNP in the MR study were significantly >10, indicating that there were no weak instrumental variables (Supplementary Table 3). Due to the number of SNP was <3, there was no values of horizontal pleiotropy in the instrumental variables (IVs) used for the genes associated with DM-PAD. The modified Cochran Q statistic demonstrated that all *p*-values were >0.05, indicating that there was no significant heterogeneity in the impact of genes and DM-PAD (Supplementary Table 4).

SMR analysis also showed consistent causal associations between genetically predicted *CYP21A2, DENND5B* and increased risk of DM-PAD: *CYP21A2* (OR: 1.49; 95%CI: 1.33–1.66; pSMR_FDR_ = 8.22 × 10^–9^), *DENND5B* (OR: 1.26; 95%CI: 1.21–1.40; pSMR_FDR_ = 2.31 × 10^–2^), while *C4A* was associated with decreased risk of DM-PAD: *C4A* (OR: 0.73; 95%CI: 0.67–0.79; pSMR_FDR_ = 3.54 × 10^–9^; Figure 3B). Generating plots of effect sizes from DM-PAD GWAS against those from all cis-eQTL in *DENND5B* (Figure 3C), *CYP21A2* (Figure 3D), and *C4A* (Figure 3E).

### COLOC and MVMR analysis

**Figure 4:**
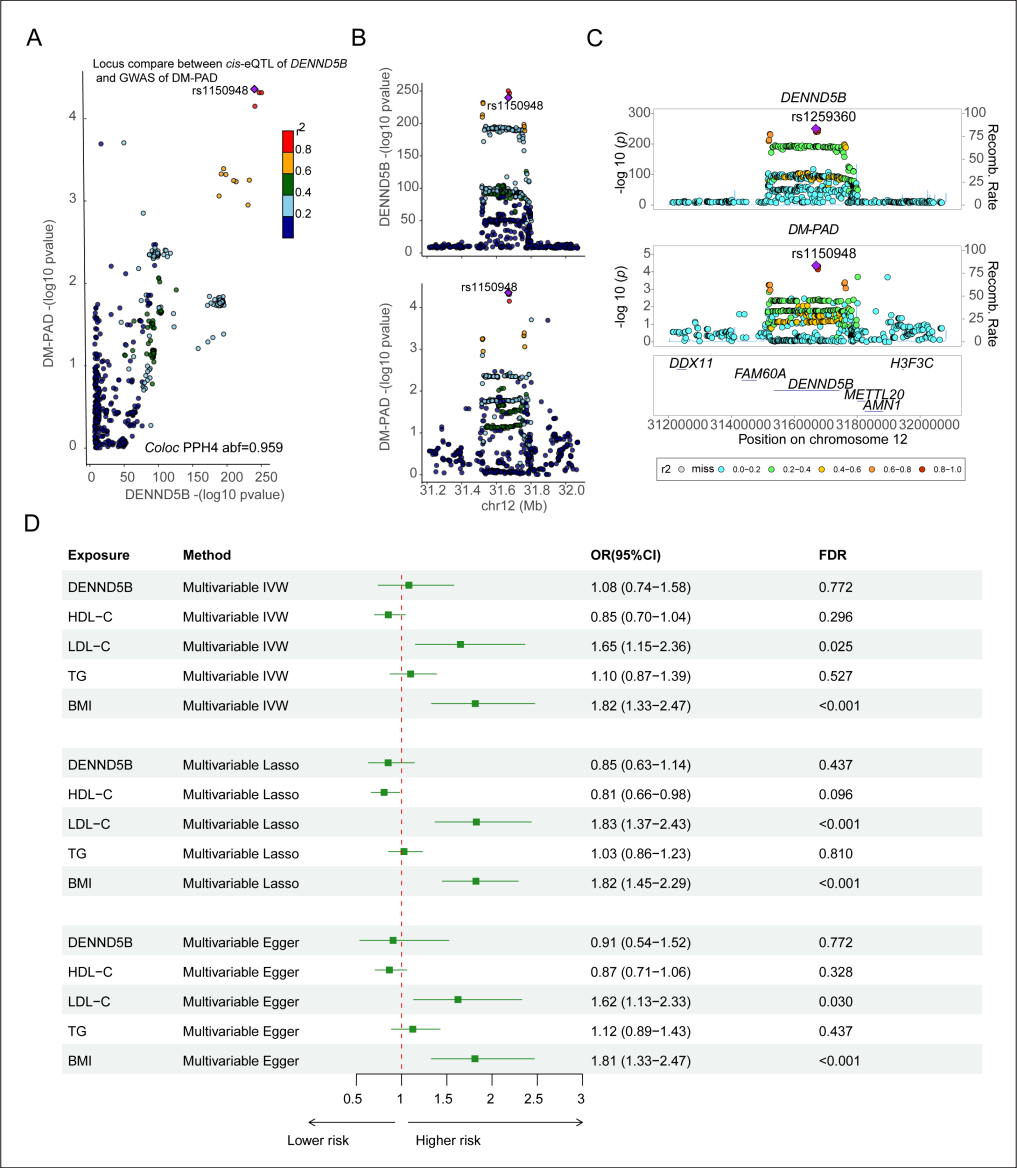
**Elucidating the association between DENND5B and DM-PAD. A.** Colocalization of genetic associations for DENND5B gene expression in the whole blood levels, and risk of DM-PAD. Each dot represents an SNP, with its color indicating the linkage disequilibrium (LD) (*r*^2^) with the GWAS lead variant, which is displayed as a purple diamond. The *p*-values from both the DM-PAD GWAS and the DENND5B gene expression analysis are compared. **B.** Genomic positions on chromosome 12 are displayed on the *x*-axis, while the –log_10_ *p*-values for SNPs from the DM-PAD GWAS (bottom) and the eQTL study for the DENND5B gene (top) are presented on the *y*-axis. **C.** Regional association plots for genetic DENND5B gene and DM-PAD GWAS data. **D.** Forest plot illustrated the MVMR results for the association between DENND5B gene and DM-PAD after statistically adjusts for other confounders including BMI and lipid traits (MVMR, multivariable Mendelian randomization; DM-PAD, diabetes mellitus–peripheral artery disease; eQTLs, expression quantitative trait locus; BMI, body mass index; HDL, high-density lipoprotein cholesterol; LDL, low-density lipoprotein cholesterol).

The most significant association was found for *rs1150948* on chromosome 12, which was highly significantly linked to an increased risk of DM-PAD (GWAS *p* = 4.393 × 10^–5^). Moreover, this variant also functioned as a highly significant eQTL of *DENND5B* in whole blood (eQTL *p* = 1.144 × 10^–240^), providing strong evidence for a causal relationship between the genetic variant and the expression of the DENND5B gene (Figure 4A, B). Furthermore, the COLOC test revealed strong evidence for colocalization between the DM-PAD trait and the *DENND5B* gene (PP.H4.abf = 0.959. However, there was no evidence for colocalization between the *CYP21A2* and *C4A* genes and DM-PAD (Supplementary Table 5). Regional association plots for the DENND5B gene and DM-PAD region containing multiple risk SNPs (Figure 4C). A forest plot displays the results of MVMR analyses testing for causal relationships between *DENND5B*, BMI, LDL-C, HDL-C, TG, and DM-PAD. The results indicate that only LDL-C (multivariable IVW, OR:1.65; 95%CI:1.15–2.36, FDR = 0.025) and BMI (multivariable IVW, OR:1.82; 95%CI:1.33–2.47, FDR < 0.001) have a causal association with DM-PAD, while DENND5B gene, does not appear to be directly involved in the development of DM-PAD (multivariable IVW, OR:1.08; 95%CI: 0.74–1.58, FDR = 0.772; Figure 4D).

## Discussion

Patients with DM-PAD exhibit a higher prevalence of infrapopliteal or tibial artery disease and vessel calcification in comparison to non-diabetic PAD patients (24,25). However, the pathogenesis and pathogenic genes of DM-PAD are still unclear, which deserves further investigation.

In this study, we systematically integrated summary-level data from GWAS of DM-PAD and expression quantitative trait locus (eQTL) studies in blood by applying SMR and two-sample MR tests. A total of 15 candidate genes have passed the SMR and HEDI test, including six genes (*BLK, LINC02356, LOC110384692, C4A, MAX*, and *RNF1*) were found to have a protective effect and nine genes (*CPEB3, KLHDC7 A, ZNF311, CDKN1 A, PSMA4, CYP21A2, DENND5B, TGFBR2*, and *FAM167 A*) were considered to be dangerous. However, among them, only three genes (*C4A, CYP21A2*, and *DENND5B*) have passed the MR test with a *p*-adjust (FDR) value <0.05. we then performed a COLOC analysis to combine the results of the GWAS and blood eQTL data for *C4A, CYP21A2*, and *DENND5B*. This analysis allowed us to determine if the genes were associated with both the trait of interest (DM-PAD) and gene expression levels in blood, and if these associations were likely due to shared underlying causal variants. The COLOC analysis showed that strong evidence for colocalization between the DM-PAD trait and the *DENND5B* gene (PP.H4abf = 0.959), indicating that the observed association between *DENND5B* expression levels in blood and DM-PAD risk is likely due to shared causal variants (rs1150948). However, there was no evidence for colocalization between the CYP21A2 and C4A genes and DM-PAD.

*DENND5B*, DENN Domain Containing 5B, promotes the exchange of GDP to GTP, converting inactive GDP-bound Rab proteins into their active GTP-bound form (26). It has been reported that DENND5B gene plays a pivotal role in facilitating the intestinal assimilation of dietary lipids in murine organisms. Furthermore, it has been observed that Dennd5b^–/–^ mice demonstrate resistance to weight gain induced by dietary intake and exhibit reduced susceptibility to PCSK9-induced hypercholesterolemia. Moreover, these mice manifest diminished atherosclerotic lesions and a reduction in hepatic lipid content when compared to their DENND5B^+/+^ counterparts (27). Another study has highlighted the significance of DENND5B in post-Golgi chylomicron secretion. This process bears implications for body composition and peripheral lipoprotein metabolism, as evidenced by research conducted in murine models and validated by two separate exome sequencing investigations in human subjects (28). In summary, DENND5B gene plays an important role in lipid metabolism and diseases related to lipid metabolism. However, its role in DM-PAD has not been reported. Our investigation has provided preliminary evidence suggesting a causal relationship between the DENND5B gene and diabetes-peripheral artery disease (DM-PAD), highlighting their significance in the etiology of this condition, which may identify novel targets for future research on DM-PAD. Furthermore, owing to the DENND5B’s intricate interplay with lipid metabolism and body mass, we conducted a MVMR analysis. Interestingly, these findings reveal that DENND5B and DM-PAD lack causative relationships when adjusted for variables such as BMI, LDL-C, HDL-C, and TG. It is suggested that DENND5B is not an independent risk factor for DM-PAD. We cautiously speculate that the DENND5B gene may be involved in the pathological process of DM-PAD by regulating lipid metabolism. However, its specific molecular mechanism requires further investigation through molecular biology experiments.

*C4A*, or complement C4A (Rodgers blood group), was demonstrated to be downregulated in patients with diabetes who showed severe coronary artery stenosis and can serve as a plasma biomarker for predicting the severity of coronary artery atherosclerosis (29). Consistent with these observations, our results suggest that *C4A* may act protective factors for DM-PAD. However, the detailed roles of *C4A* in DM-PAD have not been addressed that warrant further functional investigation.

As for CYP21A2 gene, there was no relevant literature reported the association between *CYP21A2* and atherosclerosis or DM-PAD.

It is worth noting that, in our study, *C4A* and *CYP21A2* gene passed our SMR and two sample MR test but failed our COLOC tests; however, the *p*-value of C4A and CYP21A2 gene from the SMR and two sample MR test was much smaller than that of DENND5B gene (Figure 3A, B). Therefore, the function of the *C4A* and *CYP21A2* in the development of DM-PAD should be analyzed and validated in future studies even if they failed our COLOC tests.

## Limitation

There were also several major limitations to our study. First, the SMR method cannot differentiate pleiotropic genes from causal genes. Therefore, it is important to emphasize that the association between the prioritized genes and DM-PAD must be validated through follow-up functional studies. Second, our SMR, two-sample MR, and COLOC analyses were conducted primarily using populations of European ancestry. Consequently, it is important to note that the generalizability of our findings to non-European populations and diverse ethnicities may be limited. Third, the eQTL data we used were derived exclusively from whole blood samples, data from peripheral arterial samples were not available for further validation.

## Conclusion

Taken together, our study is the first to investigate the relationship between ethnic genetic and DM-PAD risk using integrated multi-omics Mendelian randomization methods. We identified *DENND5B, C4A*, and *CYP21A2* as potential susceptibility genes for DM-PAD. Notably, our findings provide strong evidence that the association between the polymorphism *rs1150948* and the risk of DM-PAD is mediated by its effect on the expression of DENND5B. This suggests that the regulation of DENND5B could represent a potential new target for DM-PAD treatment, providing a theoretical foundation for the development of novel targeted therapies for DM-PAD patients in the future. Furthermore, the expression level of DENND5B in plasma is significantly associated with the onset and progression of DM-PAD in patients. The potential of DENND5B expression as a novel diagnostic biomarker for DM-PAD warrants further investigation and represents an emerging avenue for future research.

## Clinical Perspective

What Is New?

- Mendelian randomization provides evidence for a potentially causal relationship between DENND5B gene and diabetes–peripheral artery disease

What Are the Clinical Implications?

- These findings shedding light on the underlying mechanisms and novel target of the diabetes–peripheral artery disease in future research

## Nonstandard Abbreviations and Acronyms

**Table:** 

DM-PAD	diabetes mellitus–peripheral artery disease
eQTLs	expression quantitative trait locus
MR	Mendelian randomization
MVMR	multivariate Mendelian randomization
SMR	summary data-based Mendelian randomization
Coloc	colocalization

## Data Availability Statement

The summary-level data of the GWAS on DM-PAD was publicly available from the FinnGen consortium (https://www.finngen.fi/en). The eQTL dataset was publicly available from the eQTLGen consortium (https://www.eqtlgen.org/).

## Additional File

The additional file for this article can be found as follows:

10.5334/gh.1373.s1 Supplementary File. Tables 1 to 5.
