# Supplementary material for: DENND5B Gene Expression as a Trigger for the Development of Diabetes Mellitus–Peripheral Artery Disease: Insights from a Univariate and Multivariate Mendelian Randomization Study
Source: Glob Heart. 2024 Dec 5;19(1):91. doi: 10.5334/gh.1373 (PMC11623097; doi:10.5334/gh.1373)
Supplement: Supplementary File. — Tables 1 to 5. [file gh-19-1-1373-s1.pdf]

Supplementary Table 1. SMR analyses to identify pathogenic genes for DM-PAD using 15727 top SNP

SYMBOL	ProbeChr	topSNP	topSNP_chr	topSNP_bp	A1	A2	Freq	b_GWAS	se_GWAS	p_GWAS	b_eQTL	se_eQTL	p_eQTL	b_SMR	se_SMR	p_SMR	p_HEIDI	nsnp_HEIDI	padjust (FDR)	or (95CI)
PSMA4	15	rs931794	15	78826180	G	A	0.382704	0.0758826	0.0157166	1.38E-06	0.183504	0.00835264	5.63E-107	0.41352	0.0876909	2.41E-06	0.3348387	20	0.001506278	1.51 (1.27-1.80)
CPEB3	10	rs833385	10	93831646	G	C	0.374751	-0.0629373	0.015057	2.92E-05	-0.0928735	0.00810796	2.23E-30	0.677667	0.172581	8.61E-05	0.02934978	20	0.032833201	1.97 (1.40-2.76)
CDKN1A	6	rs12199346	6	36641546	A	C	0.208748	-0.0956879	0.0192995	7.12E-07	-0.208691	0.00919676	5.40E-114	0.458514	0.0946605	1.27E-06	0.1370627	20	0.000865668	1.58 (1.31-1.90)
MAX	14	rs998247	14	65546299	T	C	0.39165	0.0643369	0.0151892	0.000022783	-0.197013	0.00805581	4.34E-132	-0.326562	0.0782453	3.00E-05	0.2288474	20	0.014645814	0.72 (0.62-0.84)
BLK	8	rs4840568	8	11351019	A	G	0.266402	0.0707609	0.0170241	3.23E-05	-0.443265	0.00851602	0	-0.159636	0.0385284	3.42E-05	0.477594	20	0.015735103	0.85 (0.79-0.92)
RNF145	5	rs6891575	5	158630041	A	G	0.4334	0.0612223	0.0153736	0.000068248	-0.141667	0.00798474	1.98E-70	-0.432157	0.111219	0.000102067	0.1689442	20	0.036533676	0.65 (0.52-0.81)
FAM167A	8	rs4840568	8	11351019	A	G	0.266402	0.0707609	0.0170241	3.23E-05	0.746637	0.0085986	0	0.0947728	0.0228271	3.30E-05	0.6443233	20	0.015625628	1.10 (1.05-1.15)
TGFBR2	3	rs3821669	3	30694120	A	G	0.124254	0.0880431	0.0229696	0.000126579	0.393963	0.0116574	2.33E-250	0.22348	0.0586777	0.000139751	0.5381453	20	0.046471701	1.25 (1.11-1.40)
DENND5B	12	rs1259360	12	31668245	C	T	0.382704	-0.0620488	0.0152712	4.84E-05	-0.27185	0.00803215	4.27E-251	0.228247	0.0565785	5.48E-05	0.5470151	20	0.023147052	1.26 (1.12-1.40)
KLHDC7A	1	rs2992756	1	18807339	T	C	0.511928	-0.0656727	0.0151084	1.38E-05	-0.104901	0.00798542	2.03E-39	0.626043	0.151705	3.68E-05	0.03135099	20	0.016431563	1.87 (1.39-2.52)
ZNF311	6	rs115196926	6	28864100	T	C	0.084493	0.097811	0.0234483	0.000030281	0.172604	0.0170621	4.68E-24	0.566679	0.146946	0.000115085	0.08880652	20	0.039970126	1.76 (1.32-2.35)
CYP21A2	6	rs1150753	6	32059867	G	A	0.0755467	0.186213	0.025227	1.57E-13	0.468653	0.0227479	2.63E-94	0.397337	0.0571795	3.68E-12	0.2243351	20	8.22E-09	1.49 (1.33-1.66)
C4A	6	rs1269852	6	32080191	C	G	0.0755467	0.186451	0.0252223	1.44E-13	-0.587905	0.0226946	5.85E-148	-0.317145	0.0446146	1.17E-12	0.0442613	20	3.54E-09	0.73 (0.67-0.79)
LOC110384692	6	rs1269852	6	32080191	C	G	0.0755467	0.186451	0.0252223	1.44E-13	-0.587905	0.0226946	5.85E-148	-0.317145	0.0446146	1.17E-12	0.0442613	20	3.54E-09	0.73 (0.67-0.79)
LINC02356	12	rs3742004	12	111798553	G	A	0.210736	-0.0746191	0.0182121	4.18E-05	0.350588	0.0245538	2.98E-46	-0.21284	0.0540437	8.21E-05	0.2615667	20	0.032064538	0.81 (0.73-0.90)

Supplementary Table 2. Positive Mendelian Randomization (MR) results were obtained for candidate risk genes associated with DM-PAD at a significance level of FDR < 0.05

ENSEMBL	id.exposure.x	id.outcome.x	outcome.x	exposure.x	method	nsnp	b	se	pval	lo_ci	up_ci	or	or_lci95	or_uci95	estimate	SYMBOL	padjust (FDR)
ENSG00000136573	eqtl-a-ENSG00000136573	IRzB92	Peripheral atherosclerosis_R7	ENSG00000136573 || id:eqtl-a-ENSG00000136573	Inverse variance weighted	2	0.006046899	0.075605464	0.93625344	-0.142139811	0.154233608	1.006065218	0.867499962	1.16676342	1.01 (0.87-1.17)	BLK	0.93625344
ENSG00000125952	eqtl-a-ENSG00000125952	IRzB92	Peripheral atherosclerosis_R7	ENSG00000125952 || id:eqtl-a-ENSG00000125952	Inverse variance weighted	2	-0.047279527	0.126709761	0.709049845	-0.295630659	0.201071604	0.953820741	0.74406219	1.22271232	0.95 (0.74-1.22)	MAX	0.765773833
ENSG00000154319	eqtl-a-ENSG00000154319	IRzB92	Peripheral atherosclerosis_R7	ENSG00000154319 || id:eqtl-a-ENSG00000154319	MR Egger	3	0.316068106	0.148891272	0.280265296	0.024241213	0.607894999	1.371723676	1.02453742	1.836561364	1.37 (1.02-1.84)	FAM167A	0.445127235
ENSG00000163513	eqtl-a-ENSG00000163513	IRzB92	Peripheral atherosclerosis_R7	ENSG00000163513 || id:eqtl-a-ENSG00000163513	Inverse variance weighted	2	0.13828746	0.114057301	0.225344581	-0.08526485	0.361839769	1.148305594	0.918269049	1.435968837	1.15 (0.92-1.44)	TGFBR2	0.436488478
ENSG00000197935	eqtl-a-ENSG00000197935	IRzB92	Peripheral atherosclerosis_R7	ENSG00000197935 || id:eqtl-a-ENSG00000197935	Wald ratio	1	0.162130008	0.116065039	0.162446871	-0.065357468	0.389617484	1.176013122	0.936732551	1.476415933	1.18 (0.94-1.48)	ZNF311	0.436488478
ENSG00000170456	eqtl-a-ENSG00000170456	IRzB92	Peripheral atherosclerosis_R7	ENSG00000170456 || id:eqtl-a-ENSG00000170456	Inverse variance weighted	2	0.158857098	0.038000787	2.91044E-05	0.084375554	0.233338641	1.172170429	1.088037434	1.262809046	1.17 (1.09-1.26)	DENND5B	0.000157164
ENSG00000231852	eqtl-a-ENSG00000231852	IRzB92	Peripheral atherosclerosis_R7	ENSG00000231852 || id:eqtl-a-ENSG00000231852	Inverse variance weighted	2	0.288118348	0.058741513	9.34985E-07	0.172984982	0.403251714	1.333915161	1.18884825	1.49668358	1.33 (1.19-1.50)	CYP21A2	8.41486E-06
ENSG00000244731	eqtl-a-ENSG00000244731	IRzB92	Peripheral atherosclerosis_R7	ENSG00000244731 || id:eqtl-a-ENSG00000244731	Wald ratio	1	-0.304155132	0.041091176	1.34192E-13	-0.384693837	-0.223616426	0.73774641	0.680658997	0.799621789	0.74 (0.68-0.80)	C4A	3.62318E-12

Supplementary Table 3. All candidate instrumental variables (Ivs) are valid SNPs (F>10)

ENSEMBL	SYMBOL	SNP	effect_allele.exposure	other_allele.exposure	effect_allele.outcome	other_allele.outcome	beta.exposure	beta.outcome	eaf.exposure	eaf.outcome	chr.outcome	pos.outcome	gene.outcome	pval.outcome	se.outcome	outcome	ncase.outcome	ncontrol.outcome	samplesize.outcome	chr.exposure	pval.exposure	pos.exposure	se.exposure	samplesize.exposure	R2	F
ENSG00000125952	MAX	rs1354034	C	T	C	T	0.16076	0.00914436	0.618137	0.705557	3	56815721	ARHGEF3	0.580382	0.0165411	Peripheral atherosclerosis_R7	11197	225597	236794	3	8.34257E-40	56849749	0.0121754	31684	0.005472256	174.3259754
ENSG00000125952	MAX	rs7151272	G	A	G	A	-0.273663	0.0551208	0.0841608	0.0512122	14	65068367	MAX	0.107572	0.0342535	Peripheral atherosclerosis_R7	11197	225597	236794	14	9.78814E-38	65535085	0.0213137	31560	0.005196546	164.8492392
ENSG00000136573	BLK	rs2250412	A	G	A	G	-0.222324	0.00518806	0.824261	0.730224	8	11497061	BLK	0.760386	0.0170115	Peripheral atherosclerosis_R7	11197	225597	236794	8	1.64589E-46	11354570	0.0155255	31566	0.00645431	205.0472677
ENSG00000136573	BLK	rs7210990	A	C	A	C	0.0762532	0.0152957	0.481977	0.5035	17	16267450	PIGL	0.308298	0.0150134	Peripheral atherosclerosis_R7	11197	225597	236794	17	1.44571E-10	16170764	0.0118939	31569	0.001300294	41.09983394
ENSG00000154319	FAM167A	rs10503423	C	G	C	G	0.450774	0.0342416	0.360019	0.367241	8	11429572	FAM167A	0.0304684	0.0158236	Peripheral atherosclerosis_R7	11197	225597	236794	8	1E-200	11287081	0.0118046	25419	0.054253936	1458.078795
ENSG00000154319	FAM167A	rs2618467	C	T	C	T	0.296215	-0.0113642	0.846651	0.743104	8	11504734	BLK	0.513763	0.0174033	Peripheral atherosclerosis_R7	11197	225597	236794	8	1.51252E-73	11362243	0.0163286	25867	0.012562605	329.0656975
ENSG00000154319	FAM167A	rs28625526	A	C	A	C	0.308007	-0.043599	0.0371575	0.0226273	8	11548733	BLK	0.39331	0.0510749	Peripheral atherosclerosis_R7	11197	225597	236794	8	9.07194E-23	11406242	0.0313592	25867	0.003715596	96.46230676
ENSG00000163513	TGF	rs148	C	T	C	T	-0.271	-0.055	0.020	0.035	3	30681	TGFB	0.176	0.041	Peripheral	11197	225597	236794	3	8.1601	30723	0.041	26420	0.001	42.21

	BR2	641607					432	4709	6642	4491		594	R2	574	0476	atherosclerosis_R7					8E-11	086	7754		595345	317149
ENSG00000163513	TGFBR2	rs149007767	T	C	T	C	0.107344	0.00548635	0.149708	0.20582	7	50330658	IKZF1	0.768576	0.0186459	Peripheral atherosclerosis_R7	11197	225597	236794	7	1.16209E-10	50370254	0.0166571	29863	0.001388736	41.52672446
ENSG00000170456	DENND5B	rs1259360	T	C	T	C	0.39411	0.0620488	0.591459	0.582437	12	31515311	DENND5B	4.84217E-05	0.0152712	Peripheral atherosclerosis_R7	11197	225597	236794	12	1E-200	31668245	0.0116445	30928	0.035714694	1145.42098
ENSG00000170456	DENND5B	rs78671654	A	G	A	G	0.190714	0.0370949	0.0574954	0.0445911	12	31229494	OVOS2	0.316967	0.0370685	Peripheral atherosclerosis_R7	11197	225597	236794	12	7.77857E-14	31382428	0.0255169	29328	0.00190108	55.85727045
ENSG00000197935	ZNF311	rs9257445	C	G	C	G	0.142807	0.0231533	0.26128	0.289089	6	28981429	ZNF311	0.162447	0.0165749	Peripheral atherosclerosis_R7	11197	225597	236794	6	3.57355E-26	28949206	0.0134938	20648	0.005395144	111.9923571
ENSG00000231852	CYP21A2	rs2857709	G	A	G	A	0.237245	0.0383399	0.865463	0.864197	6	31565037	NFKBIL1	0.0782402	0.0217718	Peripheral atherosclerosis_R7	11197	225597	236794	6	1.13344E-42	31532814	0.0173274	4899	0.036856239	187.3915481
ENSG00000231852	CYP21A2	rs389884	G	A	G	A	0.591856	0.186667	0.103295	0.090885	6	31973120	STK19	1.34215E-13	0.0252186	Peripheral atherosclerosis_R7	11197	225597	236794	6	1E-200	31940897	0.0189109	4899	0.166625185	979.1075009
ENSG00000244731	C4A	rs389884	G	A	G	A	-0.613723	0.186667	0.103295	0.090885	6	31973120	STK19	1.34215E-13	0.0252186	Peripheral atherosclerosis_R7	11197	225597	236794	6	1E-200	31940897	0.0188614	4805	0.180559717	1058.317902

Supplementary Table 4. Heterogeneity testing results for all IVs

ENSEMBL	id.exposure	id.outcome	outcome	exposure	method	Q	Q_df	Q_pval	SYMBOL
ENSG00000170456	eqtl-a-ENSG00000170456	IRzB92	Peripheral atherosclerosis_R7	ENSG00000170456 || id:eqtl-a-ENSG00000170456	Inverse variance weighted	0.034975078	1	0.851647891	DENND5B
ENSG00000231852	eqtl-a-ENSG00000231852	IRzB92	Peripheral atherosclerosis_R7	ENSG00000231852 || id:eqtl-a-ENSG00000231852	Inverse variance weighted	2.310282998	1	0.128520649	CYP21A2

Supplementary Table 5. Results of colocalization between the C4A ,CP21A2 and DENND5B genes and DM-PAD

**Exposure**	**Outcome**	**nsnps**	**PP.H0.abf**	**PP.H1.abf**	**PP.H2.abf**	**PP.H3.abf**	**PP.H4.abf**
C4A	Peripheral_atherosclerosis_R7	1319	4.586E-152	3.68112E-12	1.2158E-140	0.975901467	0.024098533
CYP21A2	Peripheral_atherosclerosis_R7	617	1.0027E-126	3.79151E-12	2.6445E-115	0.999999305	6.94591E-07
DENND5B	Peripheral_atherosclerosis_R7	766	5.8248E-245	0.034372784	1.2562E-245	0.00645376	0.959173456
